# MicroRNA-3163 targets ADAM-17 and enhances the sensitivity of hepatocellular carcinoma cells to molecular targeted agents

**DOI:** 10.1038/s41419-019-2023-1

**Published:** 2019-10-14

**Authors:** Bin Yang, Chunping Wang, Hui Xie, Yiwu Wang, Jiagan Huang, Yihui Rong, Huixin Zhang, Huifang Kong, Yongping Yang, Yinying Lu

**Affiliations:** 1 Comprehensive liver cancer Department, The Fifth Medical Center, Chinese PLA General Hospital, Beijing, 100039 China; 2 Department of Interventional Therapy, The Fifth Medical Center, Chinese PLA General Hospital, Chinese PLA, Beijing, 100039 China; 3 Department of Disease Control and Prevention, Chinese PLA The 532nd Hospital, Huangshan, 242700 Anhui Province China

**Keywords:** Cancer therapeutic resistance, Liver cancer

## Abstract

Molecular targeted agents, such as sorafenib, remain the only choice of an antitumor drug for the treatment of advanced hepatocellular carcinoma (HCC). The Notch signaling pathway plays central roles in regulating the cellular injury/stress response, anti-apoptosis, or epithelial–mesenchymal transition process in HCC cells, and is a promising target for enhancing the sensitivity of HCC cells to antitumor agents. The ADAM metalloprotease domain-17 (ADAM-17) mediates the cleavage and activation of Notch protein. In the present study, microRNA-3163 (miR-3163), which binds to the 3′-untranslated region of ADAM-17, was screened using online methods. miRDB and pre-miR-3163 sequences were prepared into lentivirus particles to infect HCC cells. miR-3163 targeted ADAM-17 and inhibited the activation of the Notch signaling pathway. Infection of HCC cells with miR-3163 enhanced their sensitivity to molecular targeted agents, such as sorafenib. Therefore, miR-3163 may contribute to the development of more effective strategies for the treatment of advanced HCC.

## Introduction

Hepatocellular carcinoma (HCC) is one of the foremost threats to public health in China due to the high rate of hepatitis B virus infection in the Chinese population^1–3^. Regardless of the administration of anti-viral treatment, a large number of patients suffering from hepatitis B virus-related chronic liver disease eventually progress to HCC, a fatal end-stage liver disease^4–6^. Unfortunately, a large proportion of patients with HCC often suffer from advanced-stage disease (e.g., advanced HCC, Barcelona Clinic Liver Cancer stage B or C) at initial diagnosis. This subset of patients is unsuitable for surgical resection and is associated with poor clinical outcome or prognosis^7,8^. Moreover, advanced HCC is resistant to radiotherapy or cytotoxic chemotherapy, and the rapid or regressive recurrence after treatment may limit the application or efficiency of local therapies, such as transarterial chemoembolization or radiofrequency ablation^9–11^. Therefore, molecular targeted therapy plays important roles in the treatment of advanced HCC^12^. As the only first-line choice of an antitumor drug, the use of molecular targeted agents (i.e., oral administration of small molecular protein kinase inhibitors, such as sorafenib) has improved the overall survival or time to progression in patients with advanced HCC^13–15^. However, only a small proportion (20–40%) of patients with advanced HCC were initially sensitive to sorafenib. Of note, treatment with sorafenib is linked to a gradual increase in resistance^16^. Therefore, it is urgent to investigate and develop novel approaches to enhance the antitumor effects of molecular targeted therapies for the treatment of advanced HCC.

The Notch signaling pathway is a key regulator of cellular fate, survival, and cellular stress/cellular injury responses in HCC cells^17,18^. The aberrant expression of Notch protein or activation of the Notch pathway has been reported in various malignancies, such as prostate cancer, colorectal cancer, breast cancer, and especially in HCC^19–23^. During clinical treatment, radiotherapy (ionizing radiation) or chemotherapeutic agents (cellular toxicity) may function as cellular injuries to HCC cells, activating Notch. This leads to the development of stronger resistance to these antitumor strategies in HCC cells^24,25^. It has been confirmed that Notch protein is cleaved and activated by the ADAM metalloprotease domain-17 (ADAM-17). This results in the release of the Notch intracellular domain (NICD) for translocation into the nucleus to mediate the transcription of pro-survival or anti-apoptosis genes, such as Survivin, B-cell lymphoma-2, or inhibitors of apoptosis proteins (IAPs)^26–28^. Increasing evidence demonstrated that inhibition of the activation of the Notch pathway may enhance the efficiency of antitumor agents in HCC cells^29,30^. Therefore, targeting ADAM-17 may be a novel strategy for inhibiting Notch activation and enhancing the sensitivity of HCC cells to antitumor treatment. In the present study, miR-3163, a microRNA targeting the 3′iuntranslated region (3′-UTR) of ADAM-17, was identified using an online tool (miRDB database). The in-vitro or in-vivo models showed that overexpression of miR-3163 enhanced the antitumor activation of molecular targeted agents.

## Material and methods

### Patients and agents

The collection of HCC clinical specimens and methods were approved by the Ethic Committee of the Fifth Medical Center of General Hospital, Chinese People’s Liberation Army (formerly named the 302nd Hospital, Chinese People’s Liberation Army). The HCC patients provided written informed consent for the collection and usage of specimens, which were previously described (Supplementary Table 1)^31,32^. A total of 52 patients were included and real-time quantitative PCR (qPCR) was performed to examine the expression of genes in clinical specimens. The primers used in the qPCR experiments are shown in Supplementary Table 2. Lentivirus particles containing NICD, pre-miR-3163, ADAM-17, or with a mutation of miR-3163 target sequences in the 3′-UTR of ADAM-17 were constructed by Vigene Corporation (Jinan, China). The vectors containing the full-length sequences of ADAM-17 were purchased from Vigene Corporation (Jinan, China) and the vectors containing ADAM-17 with mutagenized miR-3163-binding sites were constructed by PCR methods. Hepatic cell lines: L-02 (a non-tumor hepatic cell line), MHCC97-H, or LM-3 (two highly metastatic cell lines of HCC), HepG2, Hu7, BEL-7402, or SMMC-7721 (cell lines of HCC), and MHCC97-L (a lowly metastatic cell line of HCC) were purchased from the Type Culture Collection of the Chinese Academy of Sciences (Shanghai, China) or the National Infrastructure of Cell Line (Institute of Basic Medicine, Chinese Academy of Medical Science, Beijing, China); these are the two culture collection centers of the Chinese government. Five patient-derived HCC (PDC) cell lines were provided by Dr Fan Feng at the Research Center for Clinical and Translational Medicine at the 302nd Hospital of Chinese People’s Liberation Army (Beijing, China)^33^. The cell lines were maintained in our lab under conditions, which were previously described^34,35^. Molecular targeted agents (i.e., sorafenib: catalog number S7397; regorafenib: catalog number S1178; lenvatinib: catalog number S1164; anlotinib: catalog number S8726; or apatinib: catalog number S5248) were purchased from Selleck Corporation (Houston, TX, USA). These agents (4 mg each) were dissolved in a mixture of dimethyl sulfoxide (15 μl), polyethylene glycol 400 (60 μl), and Tween80 (40 μl). Physiological saline was carefully added to the solution (agents dissolved in organic solvent) to a total volume of 20 ml^36,37^. Therefore, the concentration of agents was 0.2 mg/ml.

### Subcellular fractionation and western blotting

Subcellular fractionation methods were used to examine the subcellular distribution of NICD in HCC cells^38,39^. HCC cells that were stably infected with control miRNA or miR-3163 by using lentivirus particles were collected and homogenized using a Dounce homogenizer. For subcutaneous tumor tissue formed by HCC cells, a 200-mesh steel sieve was used to grind the tumor tissue and obtain a cell suspension. Subsequently, the cell suspension was washed with physiological saline to obtain single cells. The homogenate was centrifuged at 366 × *g* for 10 min at 4 °C to collect the nuclear sub-fraction. Subsequently, the supernatant was centrifuged again at 13,201 × *g* for 15 min at 4 °C and the final supernatant was the cytoplasmic sub-fraction. Western blotting experiments were performed following a standard protocol. The antibodies against Lamin A (catalog number ab8980), β-actin (catalog number ab205), or antibodies conjugated with horseradish peroxidase were purchased from Abcam PLC (Cambridge, UK). Moreover, the antibody of NICD (catalog number sc-373891) was obtained from Santa Cruz Corporation (Dallas, TX, USA). β-Actin was used as a cytoplasmic indicator and Lamin A was selected as the indicator of the nuclear fraction.

### Extraction of RNA samples and qPCR experiments

Extraction of RNA samples and qPCR experiments were performed according to the methods described by Liang et al.^40^ and Ji et al.^41^. Briefly, the total RNA sample of cultured HCC cells or tumor tissues was extracted and reverse-transcribed into cDNA using an RNeasy Mini kit (Qiagen, Valencia, CA, USA) according to the protocol provided by the manufacturer. The TaqMan miRNA qRT-PCR (Applied Biosystems, Foster City, CA, USA) was used to detect and quantify the miRNA expression of miR-3163 as previously described by Ji et al.^41^ and Liang et al.^40^. The relative expression level of the miRNA was calculated using the comparative cycle threshold method. Universal small nuclear RNA U6 was used as the endogenous control for the miRNAs. The sequences of the primers used for the qPCR analysis are shown in Supplementary Table 2.

### Examination of cell survival using the MTT method

Cells were cultured and collected to prepare a cell suspension. Subsequently, cells were seeded into 96-well plates (8000 cells per well). Following the full attachment of cells to the bottom of the plates, the cells were treated with the indicated concentrations of molecular targeted agents (i.e., 10, 3, 1, 0.3, 0.1, 0.03, and 0.01 μmol/l) for 48 h. Subsequently, the cells were analyzed through Thiazolyl Blue Tetrazolium Bromide [3-(4,5-dimethyl-2-thiazolyl)-2,5-diphenyl-2-H-tetrazolium bromide] (MTT) analysis following previously described methods^42^. The inhibition rate was calculated as follows: (optical density [OD] 490 nm control group − OD 490 nm administration group)/(OD 490 nm control group)^43,44^.

### In-vivo tumor model

The protocols of the animal experiments were approved by the Institutional Animal Care and Use Committee of the 302nd Hospital, Chinese People’s Liberation Army, and were performed in accordance with the UK Animals (Scientific Procedures) Act, 1986, and its associated guidelines^45^. For the subcutaneous tumor model, MHCC97-H cells infected with lentivirus particles were injected into a subcutaneous location. Following the injection (4–5 days), the mice received oral administration of molecular targeted agents every 2 days. After 3 weeks of treatment (~10 administrations), the mice were collected and the tumor volumes/tumor weights were examined. The tumor volumes were calculated as follows: tumor width × tumor width × tumor length/2^46^. The inhibition rate of molecular targeted agents was calculated as follows: [(tumor volumes of the control group) − (tumor volumes of the treatment group)]/(tumor volumes of the control group) × 100% or [(tumor weights of the control group) − (tumor weights of the treatment group)]/(tumor weights of the control group) × 100%.

For the intrahepatic migration model, MHCC97-H cells infected with lentivirus particles were injected into nude mice to form a subcutaneous tumor or into the liver via hepatic portal vein injection^47^. Following the injection (4–5 days), the mice received oral administration of molecular targeted agents every 2 days. After 3 weeks of treatment (~10 administrations), the mice were analyzed using micro positron emission tomography (^micro^PET) according to the methods described by Li et al.^48^. Subsequently, the mice were collected and the livers with nodules formed by MHCC97-H of nude mice were collected. Photographs were captured and quantitatively analyzed to determine the total amount of nodules using the Image J software (version number: 1.51j8; the National Institutes of Health, Bethesda, MD, USA), according to the methods described by Shao et al.^47^. The radioactivity in the organs and blood (i.e., radio-activation of the liver to blood) was measured using a NaI (Tl) well counter (China Atom Corporation, Beijing, China). The inhibition rate was calculated as follows: [control group relative nodule area (percentages of nodules to the total area of the liver, %) − treatment group relative nodule area]/(control group relative nodule area) × 100%; [control group relative radio-activation (the radio-activation of the liver to blood, folds) − treatment group relative radio-activation]/(control group relative radio-activation) × 100%

### Statistical analysis

Statistical analysis was performed using Bonferroni’s correction without two-way analysis of variance (SPSS software [Version Number 9.0]; IBM Corporation, Armonk, NY, USA). The half maximal inhibitory concentration (IC_50_) values of agents were calculated using the Origin software (Version Number 6.1, OriginLab Corporation, Northampton, MA, USA). A *P*-value < 0.05 denoted statistical significance.

## Results

### High endogenous expression of ADAM-17 is associated with poor prognosis in patients with advanced HCC, who received sorafenib

**Fig. 1:**
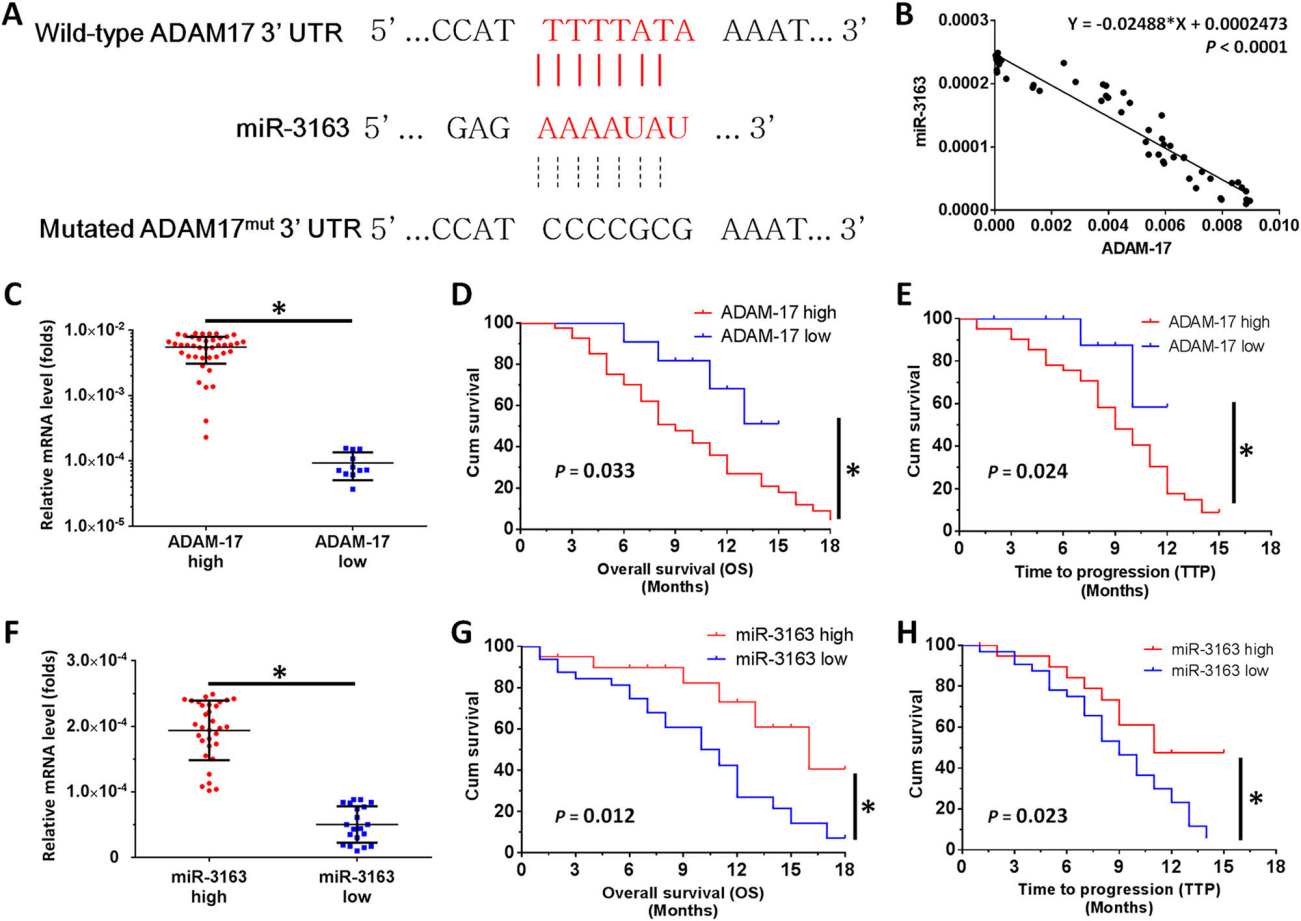
The miR-3163/ADAM-17 axis plays roles in the regulation of HCC treatment. **a** The binding site of miR-3163 in the 3′-UTR of ADAM-17. The bold and italicized fonts indicate the wild-type or mutant forms of putative miR-3163 targeting sequences. **b** The relationship between the expression level of miR-3163 and ADAM-17 in advanced HCC specimens was assessed through the Spearman’s rank correlation analysis. **c** A total of 52 patients were divided into two groups (ADAM-17-high group or ADAM-17-low group) according to the median value of ADAM-17 expression. **d**, **e** Kaplan–Meier survival curves and log-rank tests were used to analyze the OS (**d**) or TTP (**e**) in advanced HCC patients with low or high levels of ADAM-17, who received treatment with sorafenib. **f** A total of 52 patients were divided into two groups (miR-3163-high group or miR-3163-low group) according to the median value of miR-3163 expression. **g**, **h** Kaplan–Meier survival curves and log-rank tests were used to analyze the OS (**g**) or TTP (**h**) in advanced HCC patients with low or high levels of miR-3163, who received treatment with sorafenib. *P < 0.05

First, miR-3163 was identified as a microRNA targeting ADAM-17 using the online tool miRDB. As shown in Fig. 1, the bold and italicized fonts indicated the binding site of miR-3163 located in the 3′-UTR of ADAM-17 (Fig. 1a). Figure 1a also shows that mutations were introduced into the miR-3163-binding sites located in the 3′-UTR of ADAM-17. The expression of miR-3163 and ADAM-17 in HCC clinical specimens was examined to identify potential interactions. As shown in Fig. 1b, the expression of miR-3163 was negatively associated with ADAM-17 expression in the HCC specimens (*Y* = − 0.02488 × *X* + 0.0002473; *P* < 0.0001).

Subsequently, the involvement of ADAM-17 and miR-3163 in treatment with sorafenib was investigated. The endogenous level of ADAM-17 or miR-3163 was measured in clinical specimens obtained from patients with advanced HCC, who received sorafenib. By determining the median values of this expression level, the patients were divided into two groups for each factor: ADAM-17-high group or ADAM-17-low group; miR-3163-high group or miR-3163-low group. The statistical data indicated that patients in the ADAM-17-high group were linked to a poor prognosis vs. those in the ADAM-17-low group (Table 1 and Fig. 1c–e). In contrast, patients in the miR-3163-high group were associated with a better prognosis vs. those in the miR-3163-low group (Table 2 and Fig. 1f–h). The results are shown as survival curves (Fig. 1c–h), mean + 95% confidence level of overall survival, or time to progression (Tables 1 and 2), or percentage of complete response, partial response, or stable disease (Tables 1 and 2).

**Table 1:** ADAM-17 expression and clinical outcome of sorafenib treatment

	ADAM-17 mRNA expression		*P*
	High (*n* = 26)	Low (*n* = 26)	
TTP	9.0	12.0	0.024
	7.3–10.7 (M)	9.4–12.1 (M)	
OS	10.0	13.0	0.033
	6.6–11.4 (M)	10.8–14.6 (M)	
Overall response rate (PR)	0 (0%)	4 (15.38%)	
Disease control rate (PR + SD)	4 (15.38%)	9 (34.61%)	

*CR* complete remission, *M* months, *OS* overall survival, *PR* partial remission, *SD* stable of disease, *TTP* time to progress

**Table 2:** miR-3163 expression and clinical outcome of sorafenib treatment

	miR-3163 mRNA expression		*P*
	Low (*n* = 26)	High (*n* = 26)	
TTP	9.0	11.0	0.023
	7.2–10.2 (M)	9.3–13.1 (M)	
OS	11.0	16.0	0.012
	8.0–14.0 (M)	10.4–21.6 (M)	
Overall response rate (PR)	1 (3.84%)	3 (11.54%)	
Disease control rate (PR + SD)	2 (7.69%)	12 (46.15%)	

*CR* complete remission, *M* months, *OS* overall survival, *PR* partial remission, *SD* stable of disease, *TTP* time to progress

Moreover, a high level of ADAM-17 was detected in HCC cell lines compared with L-02, a non-tumor haptic cell line. In addition, the expression of ADAM-17 in LM-3 or MHCC97-H cells, two highly aggressive HCC cell lines, was much higher than that observed in other HCC cell lines (Supplementary Fig. 1). To further examine the roles of ADAM-17 or miR-362 in HCC, MHCC97-H cells—a highly aggressive HCC cell line—was infected with lentivirus particles and was injected into nude mice to form subcutaneous tumors. As shown in Supplementary Fig. 2, overexpression of ADAM-17 enhanced the subcutaneous growth of MHCC97-H cells in nude mice and decreased the antitumor effect of sorafenib on HCC cells. Transfection of miR-3163 inhibited the subcutaneous growth of MHCC97-H cells in nude mice (Supplementary Fig. 3) and ADAM-17Mut or NICD could block the effect of miR-3163 on the subcutaneous growth of MHCC97-H cells (Supplementary Fig. 3). Therefore, the miR-3163/ADAM-17 axis plays an important role in the regulation of HCC.

### miR-3163 inhibits the expression of ADAM-17 by targeting the 3′-UTR of ADAM-17 mRNA

**Fig. 2:**
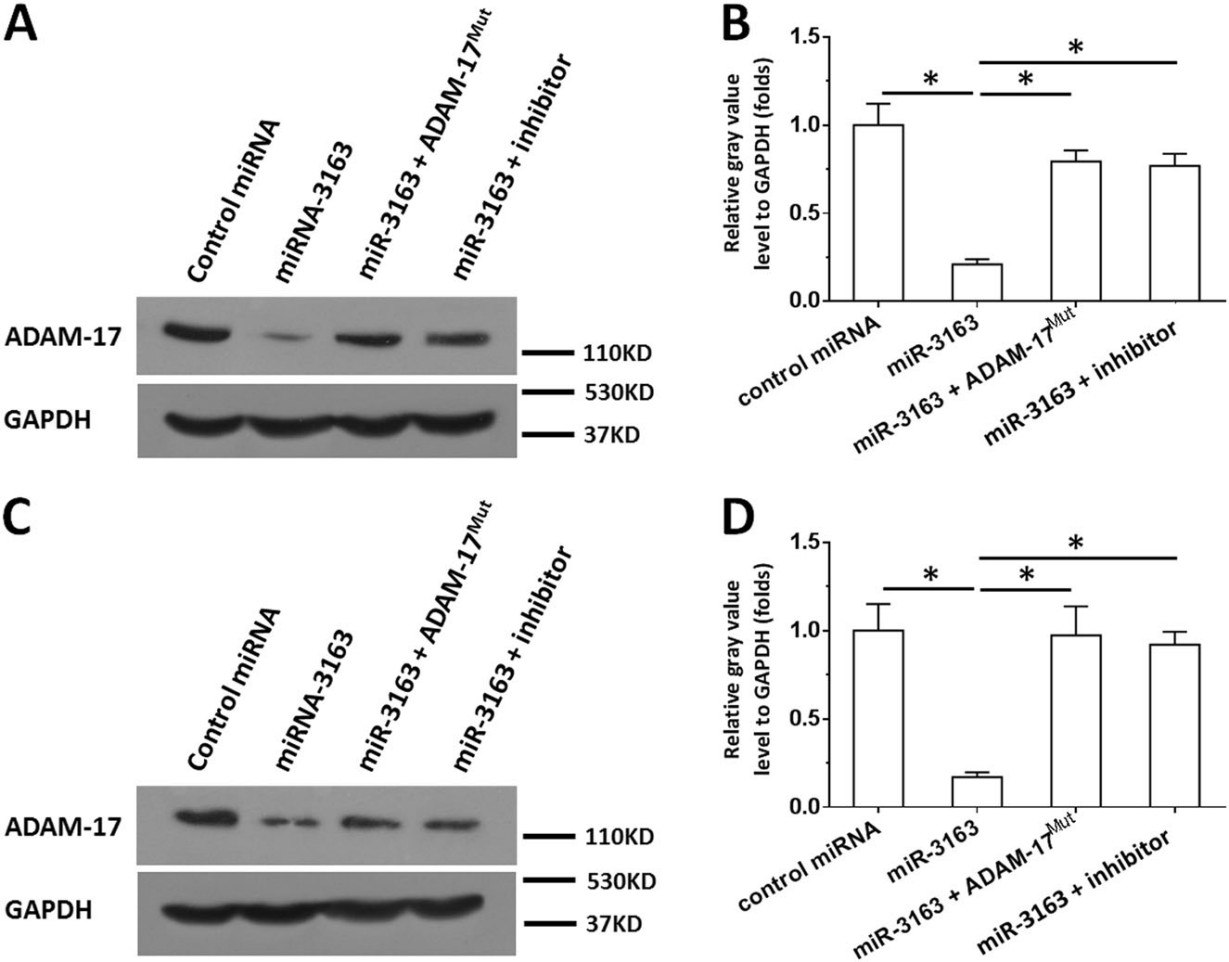
miR-3163 suppresses the expression of ADAM-17. MHCC97-H (**a**, **b**) or LM-3 (**c**, **d**) cells transfected with control miRNA, miR-3163, miR-3163 + ADAM-17^Mut^ (ADAM-17 with mutated miR-3163 binding sites), or miR-3163 + its inhibitor, were collected for western blotting experiments. The protein level of ADAM-17 or GAPDH was examined using antibodies. GAPDH was selected as loading control. The results are shown as images of western blotting (**a**, **c**) or quantitative analysis (**b**, **d**). *P < 0.05

The expression vectors of ADAM-17 with mutated miR-3163-targeted sequences were also constructed to confirm whether miR-3163 targets ADAM-17. As shown in Fig. 2, compared with the control miRNA, miR-3163 significantly repressed the expression of ADAM-17 in MHCC97-H (Fig. 2a, b) or LM-3 (Fig. 2c, d). This effect was not observed for ADAM-17^Mut^, which contains a mutation in the miR-3163-binding sites. Transfection of the miR-3163 inhibitor almost blocked the decreasing effect of miR-3163 on the expression of ADAM-17 (Fig. 2). Moreover, the interaction between the 3ʹ-UTR of ADAM-17 and miR-3163 was confirmed through luciferase experiments (Supplementary Figs. 4 and 5). Therefore, ADAM-17 may be a target of miR-3163. It is suggested that miR-3163 may repress the expression of ADAM-17 in HCC cells by targeting the 3′-UTR of ADAM-17.

### Overexpression of miR-3163 inhibits the activation of the Notch signaling pathway

**Fig. 3:**
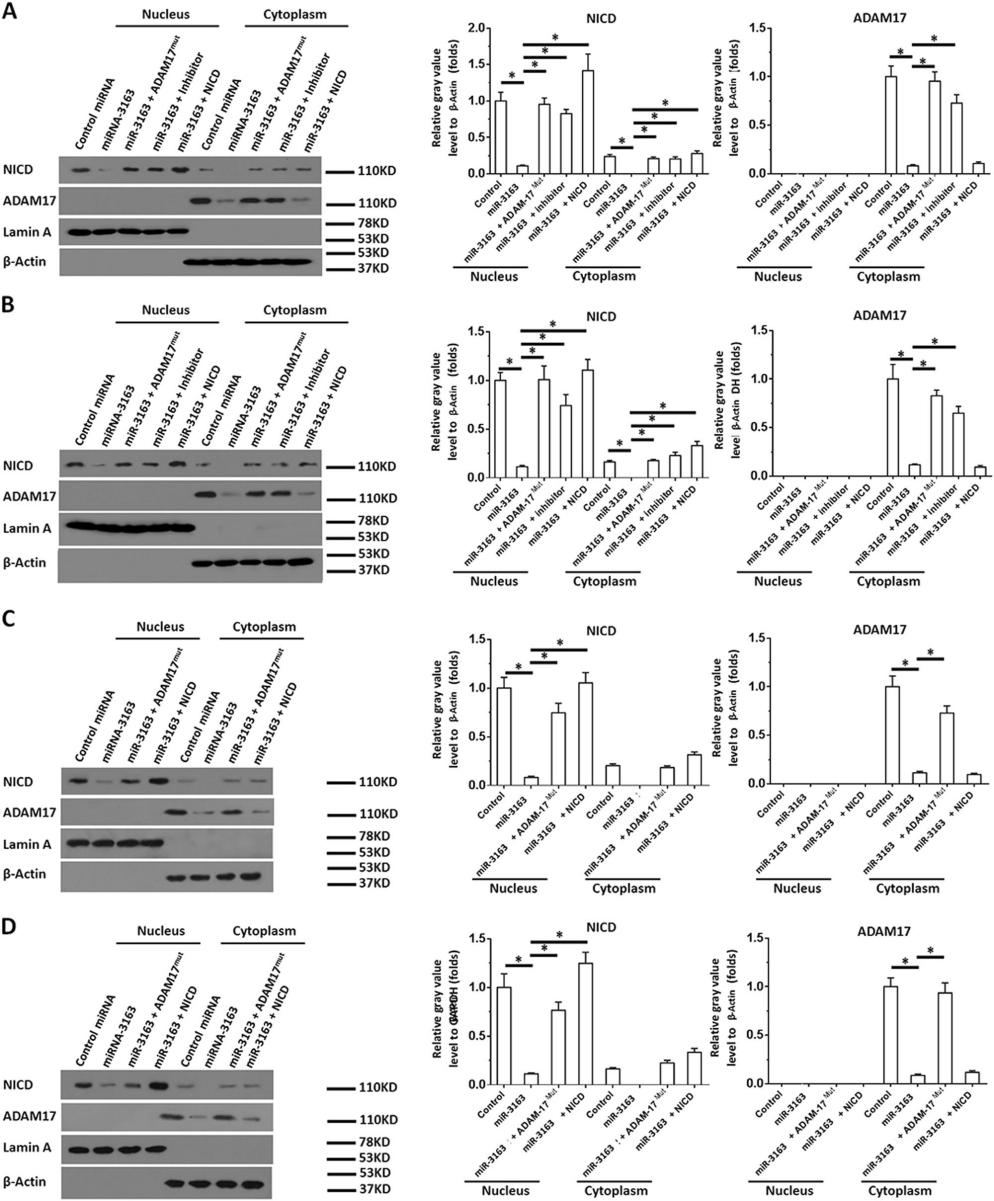
miR-3163 inhibits the accumulation of NICD in the nuclear sub-fraction of HCC cells. MHCC97-H cells (**a**, **c**) or LM-3 cells (**b**, **d**) transfected with vectors (control miRNA, miR-3163, miR-3163 + ADAM-17^Mut^, or miR-3163 + NICD) were analyzed in the subcellular fractionation experiments. The accumulation of ADAM-17 or NICD in cultured cells (**a**, **b**) or single cells separated from subcutaneous tumor tissues (**c**, **d**) formed by MHCC97-H (**c**) or LM-3 (**d**) cells was examined using antibodies. Lamin A, a nuclear skeleton protein, was used as an indicator of the nuclear sub-fraction; β-actin was used as an indicator of the cytoplasmic sub-fraction. The results are shown as images of western blotting or quantitative analysis. *P < 0.05

The accumulation of NICD in the nucleus was examined to further identify the effect of miR-3163 on the activation of the Notch signaling pathway. As shown in Fig. 3, overexpression of miR-3163 significantly inhibited the expression of ADAM-17 in the cytoplasm of MHCC97-H (Fig. 3a) or LM-3 cells (Fig. 3b), and decreased the accumulation of the NICD of Notch protein in the nucleus of MHCC97-H (Fig. 3a) or LM-3 (Fig. 3b) cells. Transfection of ADAM-17^Mut^ or the inhibitor of miR-3163 almost blocked the inhibitory effect of miR-3163 on the cleavage of Notch protein and the accumulation of NICD in the nucleus (Fig. 3a, b). Subsequently, HCC cells infected with lentivirus particles were injected into nude mice to form subcutaneous tumors and the accumulation of NICD in the nucleus of single cells. As shown in Fig. 3, overexpression of miR-3163 significantly inhibited the expression of ADAM-17 in the cytoplasm of MHCC97-H (Fig. 3c) or LM-3 cells (Fig. 3d) separated from subcutaneous tumors. Moreover, it decreased the accumulation of the NICD of Notch protein in the nucleus of MHCC97-H (Fig. 3c) or LM-3 (Fig. 3d) cells. Transfection of ADAM-17^Mut^ almost blocked the inhibitory effect of miR-3163 on the cleavage of the Notch protein and the accumulation of NICD in the nucleus of cells separated from subcutaneous tumors (Fig. 3c, d).

**Fig. 4:**
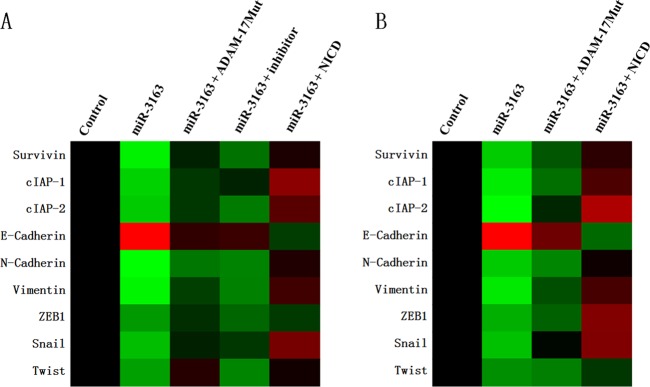
miR-3163 inhibits the activation of the Notch signaling pathway. MHCC97-H cells transfected with vectors (control miRNA, miR-3163, miR-3163 + ADAM-17^Mut^, or miR-3163 + NICD) were analyzed in the qPCR experiments. The expression of Survivin, cIAP-1, cIAP2, E-Cadherin, N-Cadherin, Vimentin, ZEB1, Snail, or Twist in cultured cells (**a**) or subcutaneous tumor tissues formed by MHCC97-H (**b**) was examined through qPCR. The results are shown as a heat-map according to the relative mRNA level of Survivin, cIAP-1, cIAP-2, E-Cadherin, N-Cadherin, Vimentin, ZEB1, Snail, or Twist

Subsequently, the expression of the following downstream factors of the Notch pathway was determined: epithelial–mesenchymal transition (EMT)-related factors (E-cadherin, an epithelial indicator; N-cadherin or Vimentin, two mesenchymal indicators; EMT-related transcription factors, ZEB1 or Snail), and pro-survival/anti-apoptosis-related factors (Survivin, cellular IAP-1 (cIAP-1), or cIAP2). As shown in Fig. 4, overexpression of miR-3163 inhibited the expression of N-Cadherin, Vimentin, Survivin, cIAP-1, cIAP-2, Snail, or ZEB1. In contrast, it enhanced the expression of E-Cadherin. Transfection of ADAM-17^Mut^ (Fig. 4a, b) or the inhibitor of miR-3163 (Fig. 4a) almost blocked the effect of miR-3163. Similar results were obtained in cultured MHCC97-H cells (Fig. 4a) or subcutaneous tumors (Fig. 4b) formed by MHCC97-H cells. Therefore, miR-3163 inhibits the activation of the Notch signaling pathway by repressing the expression of ADAM-17.

### Overexpression of miR-3163 enhances the sensitivity of HCC cells to molecular targeted agents

Subsequently, the effect of miR-3163 on the antitumor activity of molecular targeted agents was examined. As shown in Table 3, overexpression of miR-3163 enhanced the sensitivity of MHCC97-H cells to sorafenib. Of note, the IC_50_ values of sorafenib decreased from 1.04 ± 0.05 μmol/l to 0.10 ± 0.01 μmol/l. Transfection of ADAM-17^Mut^ reduced the effect of miR-3163, with the IC_50_ values of sorafenib increasing from 0.10 ± 0.01 μmol/l to 0.77 ± 0.14 μmol/l (Table 3). Similar results were obtained in LM-3 cells (Table 3). Subsequently, the effect of miR-3163 on the sensitivity of PDC cells to molecular targeted agents was examined in patient-derived cell lines. As shown in Table 4, overexpression of miR-3163 enhanced the sensitivity of five PDCs to the molecular targeted agents (i.e., sorafenib, regorafenib, lenvatinib, anlotinib, or apatinib).

**Table 3:** miR-3163 enhances the sensitivity of HCC cells to sorafenib

Cell lines	control miRNA	miR-3163	miR-3163 + ADAM-17^Mut^
	IC_50_ values of sorafenib on HCC cells’ survival		
MHCC97-H	1.04 ± 0.05	0.10 ± 0.01	0.77 ± 0.14
LM-3	0.95 ± 0.35	0.14 ± 0.07	0.89 ± 0.20

**Table 4:** miR-3163 enhanced the antitumor effect of sorafenib on cultured HCC cells’ surviving

PDCs	Groups	Sorafenib	Regorafenib	Lenvatinib	Anlotinib	Apatinib
		IC_50_ values (μmol/L) of molecular targeting agents on cultured HCC cells’ surviving				
No. 1	Control	1.46 ± 0.10	1.60 ± 0.16	0.73 ± 0.41	1.83 ± 0.33	1.98 ± 0.86
	miR-3163	0.33 ± 0.11	0.61 ± 0.08	0.12 ± 0.02	0.40 ± 0.14	0.99 ± 0.63
	miR-3163 + ADAM-17^Mut^	1.55 ± 0.08	1.58 ± 0.52	0.67 ± 0.20	1.55 ± 0.58	1.91 ± 0.11
No. 2	Control	1.51 ± 0.52	1.68 ± 0.45	0.36 ± 0.11	1.65 ± 0.59	1.59 ± 0.29
	miR-3163	0.28 ± 0.05	0.57 ± 0.24	0.07 ± 0.01	0.52 ± 0.08	0.54 ± 0.07
	miR-3163 + ADAM-17^Mut^	1.44 ± 0.85	1.86 ± 0.34	0.33 ± 0.04	1.48 ± 0.62	1.74 ± 0.43
No. 3	Control	1.24 ± 0.38	1.61 ± 0.09	0.85 ± 0.07	3.81 ± 0.53	0.99 ± 0.16
	miR-3163	0.68 ± 0.45	0.48 ± 0.06	0.22 ± 0.13	1.62 ± 0.44	0.23 ± 0.06
	miR-3163 + ADAM-17^Mut^	1.35 ± 0.26	1.40 ± 0.11	0.74 ± 0.20	2.84 ± 0.74	0.75 ± 0.15
No. 4	Control	2.39 ± 0.44	1.96 ± 0.33	1.10 ± 0.33	2.73 ± 0.98	2.16 ± 0.44
	miR-3163	0.98 ± 0.19	0.22 ± 0.04	0.58 ± 0.09	0.87 ± 0.30	0.90 ± 0.54
	miR-3163 + ADAM-17^Mut^	1.88 ± 0.69	1.63 ± 0.30	1.36 ± 0.68	2.07 ± 0.36	1.93 ± 0.84
No. 5	Control	3.10 ± 0.46	2.62 ± 0.64	1.66 ± 0.40	2.81 ± 0.10	2.74 ± 0.71
	miR-3163	1.27 ± 0.51	0.79 ± 0.28	0.40 ± 0.07	0.98 ± 0.56	1.22 ± 0.28
	miR-3163 + ADAM-17^Mut^	3.06 ± 0.47	1.70 ± 0.53	1.47 ± 0.88	1.44 ± 0.47	2.53 ± 0.21

*PDCs* patients-derived HCC cell lines

**Fig. 5:**
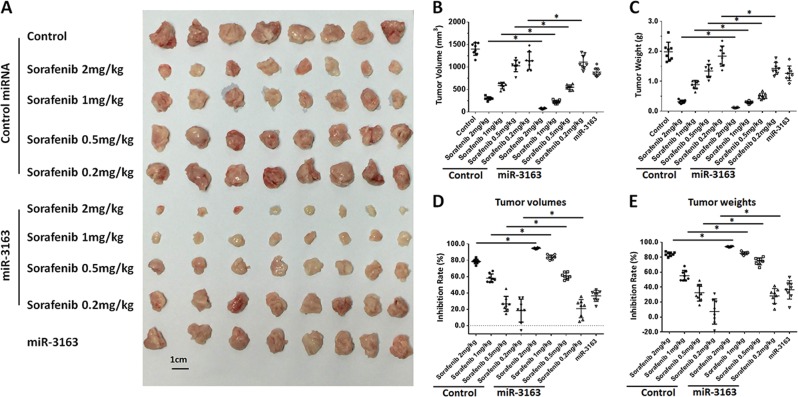
miR-3163 enhances the antitumor effect of sorafenib on the subcutaneous growth of MHCC97-H cells. MHCC97-H cells transfected with vectors (control miRNA or miR-3163) were injected into nude mice to form subcutaneous tumors. The mice received oral administration of indicated concentrations of sorafenib and were harvested to collect tumor tissues. The results are shown as images of subcutaneous tumor tissues (**a**), tumor volumes (**b**), tumor weights (**c**), inhibition rates according to tumor volumes (**d**), or inhibition rates according to tumor weights (**e**). *P < 0.05

**Fig. 6:**
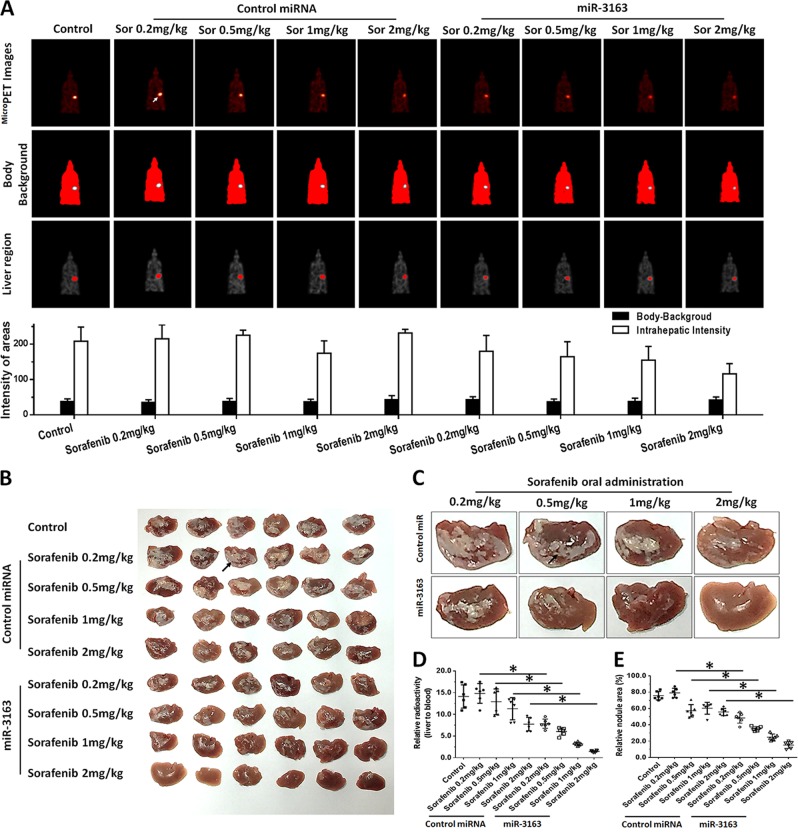
miR-3163 enhances the antitumor effect of sorafenib on the intrahepatic growth of MHCC97-H cells in the liver of nude mice. MHCC97-H cells transfected with vectors (control miRNA or miR-3163) were injected into the liver of nude mice through hepatic portal vein injection. The mice received oral administration of indicated concentrations of sorafenib. After treatment, the mice underwent ^micro^PET screening and were harvested to collect tumor tissues. The results are shown as images of micro-PET or quantitative analysis (**a**), images of livers with lesions (**b**), represented images of livers with lesions from each group (**c**), relative radio-activation of livers (**d**), or the relative area of lesions (**e**). *P < 0.05

**Fig. 7:**
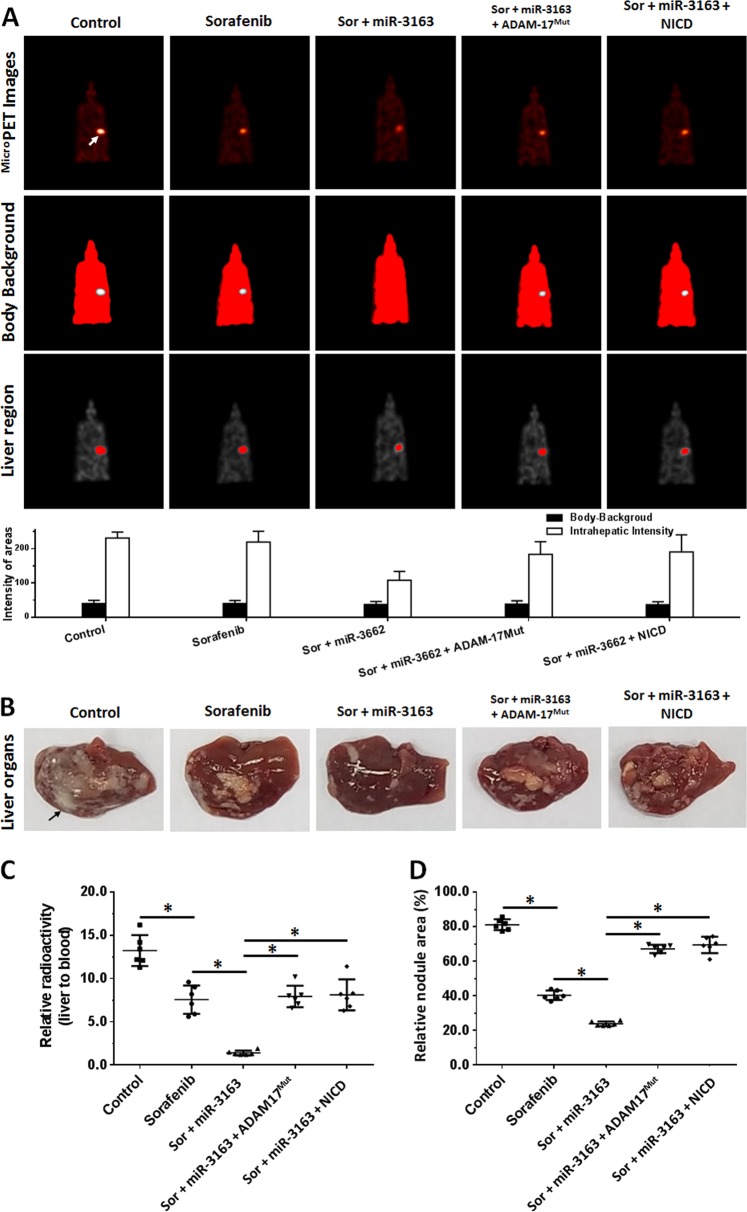
miR-3163 enhances the antitumor effect of sorafenib on the intrahepatic growth of MHCC97-H cells in the liver of nude mice by targeting ADAM-17. MHCC97-H cells transfected with vectors (control miRNA, miR-3163, miR-3163 + ADAM-17^Mut^, or miR-3163 + NICD) were injected into the liver of nude mice through hepatic portal vein injection. Mice received oral administration of 2 mg/kg dose of sorafenib. After treatment, the mice underwent ^micro^PET screening and were harvested to collect tumor tissues. The results are shown as images of micro-PET or quantitative analysis (**a**), images of livers with lesions (**b**), represented images of livers with lesions from each group (**c**), relative radio-activation of livers (**d**), or the relative area of lesions (**e**). *P < 0.05

**Fig. 8:**
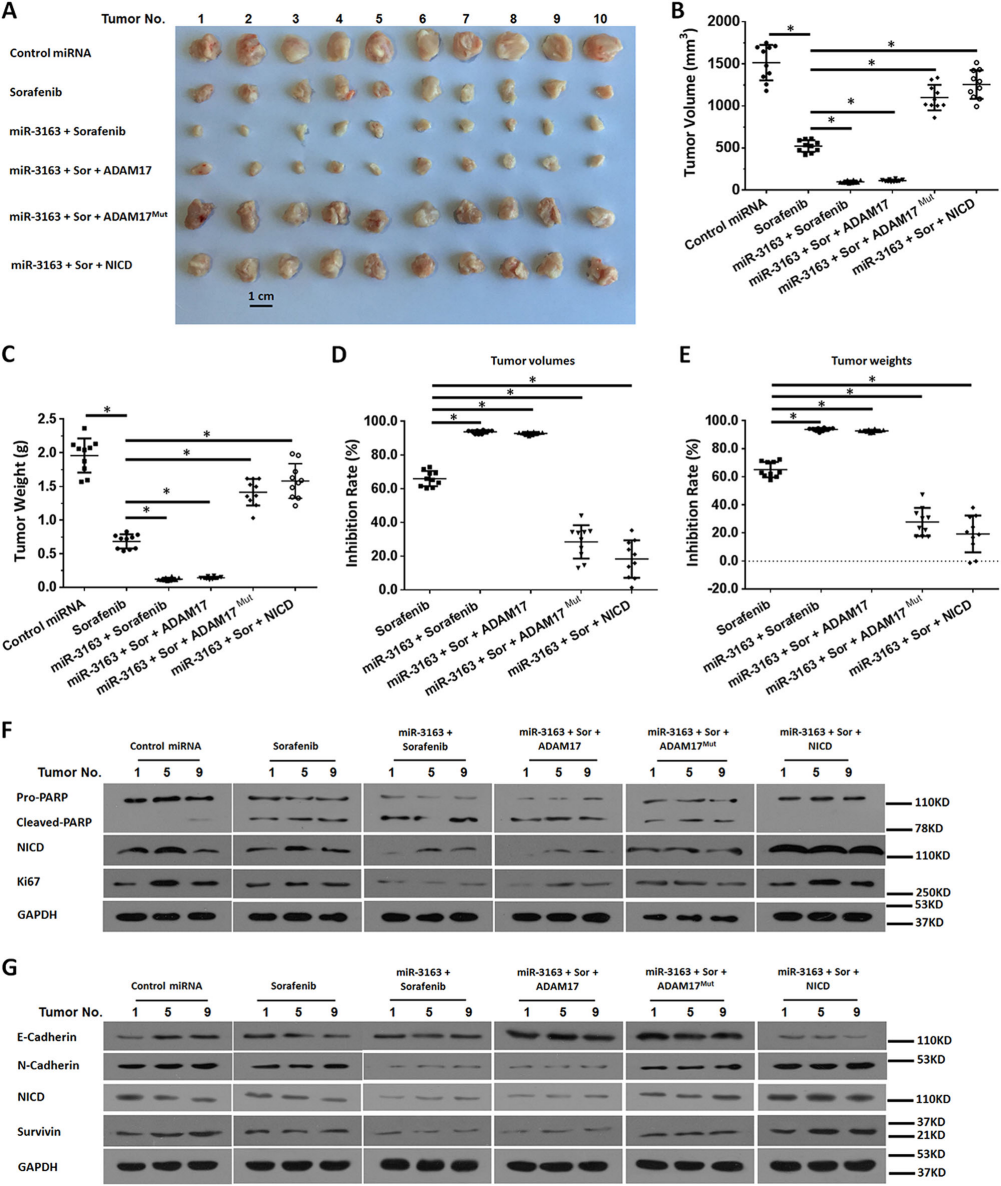
miR-3163 enhances the antitumor effect of sorafenib on the subcutaneous growth of MHCC97-H cells by targeting ADAM-17. MHCC97-H cells transfected with vectors (control miRNA, miR-3163, miR-3163 + ADAM-17, miR-3163 + ADAM-17^Mut^, or miR-3163 + NICD) were injected into nude mice to form subcutaneous tumors. The mice received oral administration of 2 mg/kg dose of sorafenib and were harvested to collect tumor tissues. The results are shown as images of subcutaneous tumor tissues (**a**), tumor volumes (**b**), tumor weights (**c**), inhibition rates according to tumor volumes (**d**), or inhibition rates according to tumor weights (**e**). The expression level of downstream factors Notch pathways: pro-survival factors (**f**) or EMT-related factors (**g**) in the represented subcutaneous tumors (the No. 1, 5 and 9 of tumors) of Fig. 8 were examined by western blotting experiments (**f**, **g**). *P < 0.05

To further examine the effect of miR-3163 on the antitumor activity of sorafenib, MHCC97-H cells were seeded into nude mice to form subcutaneous HCC tumors. As shown in Fig. 5, oral administration of sorafenib inhibited the subcutaneous growth of MHCC97-H cells. Overexpression of miR-3163 enhanced the sensitivity of HCC cells to sorafenib. Subsequently, the intrahepatic migration model was applied. As shown in Fig. 6, injection of MHCC97-H cells into the liver of nude mice via portal vein injection resulted in the formation of multiple disseminated lesions. Notably, the intrahepatic growth could be identified through ^micro^PET. Oral administration of sorafenib inhibited the images of ^micro^PET in the liver of nude mice and the area of lesions in the liver (Fig. 6). Overexpression of miR-3163 enhanced the antitumor effect of sorafenib on the intrahepatic growth of MHCC97-H cells (Fig. 6). Moreover, the specificity of miR-3163 on sorafenib was examined. As shown in Fig. 7 and Fig. 8, the expression of ADAM-17^Mut^ or NICD decreased the effect of miR-3163 on sorafenib. Similar results were obtained from PDCs: miR-3163 enhanced the sensitivity of PDCs to molecular targeted agents by targeting ADAM-17 (Table 5). To examine the specificity of miR-3163′ function, the expression level of downstream factors Notch pathways, pro-survival factors or EMT-related factors in the subcutaneous tumors of Fig. 8 were examined by western blotting experiments.

**Table 5:** miR-3163 enhanced the antitumor effect of sorafenib on HCC cells’ intrahepatic migration

PDCs	Groups	Sorafenib	Regorafenib	Lenvatinib	Anlotinib	Apatinib
		IC_50_ values (mg/kg) of molecular targeting agents on HCC cells’ intrahepatic migration				
No. 1	Control	~2	1.75 ± 0.59	1.20 ± 0.56	1.83 ± 0.23	-
	miR-3163	0.29 ± 0.09	0.38 ± 0.11	0.11 ± 0.07	0.83 ± 0.79	0.55 ± 0.15
	miR-3163 + ADAM-17^Mut^	1.89 ± 0.12	~2	1.51 ± 0.77	1.88 ± 0.64	1.65 ± 0.44
No. 2	Control	1.68 ± 0.19	1.20 ± 0.67	0.56 ± 0.06	-	-
	miR-3163	0.93 ± 0.33	0.44 ± 0.06	0.24 ± 0.07	1.84 ± 0.54	1.16 ± 0.75
	miR-3163 + ADAM-17^Mut^	1.83 ± 0.23	1.18 ± 0.54	0.72 ± 0.39	-	-
No. 3	Control	1.96 ± 0.74	-	1.04 ± 0.82	-	~2
	miR-3163	0.31 ± 0.01	0.98 ± 0.64	0.76 ± 0.20	1.16 ± 0.26	0.43 ± 0.05
	miR-3163 + ADAM-17^Mut^	1.33 ± 0.20	-	0.95 ± 0.32	-	1.84 ± 0.15
No. 4	Control	-	-	1.69 ± 0.64	-	-
	miR-3163	0.98 ± 0.09	0.30 ± 0.23	0.73 ± 0.29	1.02 ± 0.28	1.89 ± 0.62
	miR-3163 + ADAM-17^Mut^	1.82 ± 0.43	-	-	-	-
No. 5	Control	-	-	~2	-	-
	miR-3163	1.66 ± 0.35	0.85 ± 0.21	0.53 ± 0.05	1.70 ± 0.30	~2
	miR-3163 + ADAM-17^Mut^	-	-	-	-	-

*PDCs* patients-derived HCC cell lines

## Discussion

In the present study, miR-3163 was identified as a microRNA potentially targeting ADAM-17. Overexpression of miR-3162 through infection lentivirus particles inhibited the cleavage of Notch protein and enhanced the sensitivity of HCC cells to molecular targeted agents such as sorafenib. The effect of miR-3163 on the Notch signaling pathway or sensitivity of HCC cells to sorafenib was almost blocked by transfection of mutated ADAM-17, the inhibitor of miR-3163, or NICD. This confirmed the effect of miR-3163 on ADAM-17 and the sensitivity of HCC cells to molecular targeted drugs by inhibiting the expression of ADAM-17. In addition, it confirmed that the miR-3163/ADAM-17 axis acts through the Notch signaling pathway. Therefore, our results indicated that miR-3163 may enhance the sensitivity of HCC cells to sorafenib by inhibiting the cleavage of Notch protein. In addition to screening for miRNAs targeting ADAM-17, there are other strategies: (1) Jia et al.^49^ used rhamnetin to inhibit the activation of the Notch signaling pathway and enhance the sensitivity of HCC cells to sorafenib by enhancing miR-34a, which targets Notch protein; (2) Zhang et al.^50^ identified a novel inhibitor of ADAM-17; and (3) the inhibitors of the presenilin-dependent gamma secretase complex may also be useful in the treatment of HCC^51–54^.

Moreover, our results showed that miR-3163 inhibited the EMT process in HCC cells. It is established that the EMT process in cancer cells is associated with poor patient survival. Mechanism data indicated that the EMT is a key step in the progression of cancer and participates in metastasis^55^. During the EMT process, the adhesion feature of cancer cells is decreased (e.g., E-cadherin loss). Furthermore, mesenchymal markers (i.e., Vimentin or N-Cadherin) decrease the polarity of cancer cells and accelerate migration and invasion^56,57^. Recently, the EMT process has been proposed as an important regulator of drug resistance^58–60^. Accumulating data have confirmed that mechanisms of resistance to sorafenib may involve the EMT and the Notch signaling pathway is a key regulator of the EMT process^61–63^. In this study, miR-3163 significantly inhibited the EMT process in HCC cells. This means that a decrease in the expression of ADAM-17 may inhibit the activation of the Notch signaling pathway, and enhance the sensitivity of HCC cells to antitumor agents by inhibiting the EMT process. In addition to EMT, we also investigated the expression of other cell-promoting and anti-apoptotic Notch downstream proteins, including Survivin, cIAP-1, and cIAP-2^25^. Downregulation of the activity of the Notch signaling pathway by various pathways can reduce the resistance of cells to various damaging factors. This increases the sensitivity of cells to molecular targeted drugs and offers safer and more effective treatments (i.e., cytotoxic chemotherapy drugs and radiation therapy)^64–67^.

Furthermore, patient-derived tumor cells are an important model of pharmacologically relevant research that reflects the actual conditions of patients^68,69^. Constructing appropriate research models, especially animal models, contributes to the development of relevant research and provides a basis for predicting patient sensitivity and prognosis in patients who received treatment. This study used a variety of tumor animal models, including subcutaneous tumor models and intrahepatic tumor models in nude mice. The former is a common model used in oncology research. Hepatic portal vein injection was used to inoculate HCC cells into the liver of nude mice, simulating the recurrence or metastasis of HCC cells in patients. In addition Meng et al.^70–72^ established a research model for the invasion of malignant tumor cells in the liver of nude mice. Li et al.^73^ developed a breast cancer lung metastasis model in nude mice. In the future, we plan to establish new tumor models for more in-depth research. In terms of antitumor agents, this study not involved several molecular targeted drugs: sorafenib, regorafenib, lenvatinib, anlotinib, and apatinib. Regorafenib is a new secondary-line therapy option for advanced HCC developed by Bayer Corporation (Leverkusen, Nordrhein-Westfalen, Germany), whereas lenvatinib is a first-line therapy for HCC developed by Eisai Official Corporate (Tokyo, Japan)^74,75^. Anlotinib and apatinib are molecular targeted drugs developed by Chinese manufacturers (HENGRUI Medicine, Lian-yung-gang City, Jiangsu Province, China, or CHIATAI Tianqing Corporation, Nanjing City, Jiangsu Province, China)^76,77^. The mechanism of action of these drugs is similar. In the future, clinical studies investigating the use of anlotinib and apatinib for the treatment of advanced HCC may also be performed. This study found that the antitumor effect of lenvatinib may be superior to that of several other molecular targeted drugs. This provides a reference for future research.

## Supplementary information

Supplemental Table 2

Supplemental Table 1

Supplemental Figure 1

Supplemental Figure 2

Supplemental Figure 3

Supplemental Fgure 4

Supplememental Figure 5

Supplementary figures legends

## References

[1] Polaris Observatory Collaborators. (2018). Global prevalence, treatment, and prevention of hepatitis B virus infection in 2016: a modelling study. Lancet Gastroenterol. Hepatol..

[2] Wang FS (2014). The global burden of liver disease: the major impact of China. Hepatology.

[3] Zhang S, Wang F, Zhang Z (2017). Current advances in the elimination of hepatitis B in China by 2030. Front. Med..

[4] Forner A, Reig M, Bruix J (2018). Hepatocellular carcinoma. Lancet.

[5] Chen W (2016). Cancer statistics in China, 2015. CA Cancer J. Clin..

[6] Bray F (2018). Global cancer statistics 2018: GLOBOCAN estimates of incidence and mortality worldwide for 36 cancers in 185 countries. CA Cancer J. Clin..

[7] Feng F (2018). Which is the best combination of TACE and Sorafenib for advanced hepatocellular carcinoma treatment? A systematic review and network meta-analysis. Pharmacol. Res..

[8] Xie H (2017). What is the best combination treatment with transarterial chemoembolization of unresectable hepatocellular carcinoma? a systematic review and network meta-analysis. Oncotarget.

[9] Boland P, Wu J (2018). Systemic therapy for hepatocellular carcinoma: beyond sorafenib. Chin. Clin. Oncol..

[10] Meyer T (2018). Treatment of advanced hepatocellular carcinoma: beyond sorafenib. Lancet Gastroenterol. Hepatol..

[11] Kim DW, Talati C, Kim R (2017). Hepatocellular carcinoma (HCC): beyond sorafenib-chemotherapy. J. Gastrointest. Oncol..

[12] Wei L (2019). Novel urokinase-plasminogen activator inhibitor SPINK13 inhibits growth and metastasis of hepatocellular carcinoma in vivo. Pharmacol. Res..

[13] Finn RS (2018). Therapies for advanced stage hepatocellular carcinoma with macrovascular invasion or metastatic disease: a systematic review and meta-analysis. Hepatology.

[14] Llovet JM (2008). Sorafenib in advanced hepatocellular carcinoma. N. Engl. J. Med..

[15] Cheng AL (2009). Efficacy and safety of sorafenib in patients in the Asia-Pacific region with advanced hepatocellular carcinoma: a phase III randomised, double-blind, placebo-controlled trial. Lancet Oncol..

[16] Zhu YJ (2017). New knowledge of the mechanisms of sorafenib resistance in liver cancer. Acta Pharmacol. Sin..

[17] Chatterjee S, Sil PC (2019). Targeting the crosstalks of Wnt pathway with Hedgehog and Notch for cancer therapy. Pharmacol. Res..

[18] Butti R (2019). Breast cancer stem cells: biology and therapeutic implications. Int. J. Biochem. Cell Biol..

[19] Wang X (2018). Upregulation of lncRNA PlncRNA-1 indicates the poor prognosis and promotes glioma progression by activation of Notch signal pathway. Biomed. Pharmacother..

[20] Gueron G (2018). Game-changing restraint of Ros-damaged phenylalanine, upon tumor metastasis. Cell Death Dis..

[21] Kumar S (2019). Estrogen-dependent DLL1-mediated Notch signaling promotes luminal breast cancer. Oncogene.

[22] Martins-Neves SR, Cleton-Jansen AM, Gomes CMF (2018). Therapy-induced enrichment of cancer stem-like cells in solid human tumors: where do we stand?. Pharmacol. Res..

[23] Cianciosi D (2018). Targeting molecular pathways in cancer stem cells by natural bioactive compounds. Pharmacol. Res..

[24] Sosa Iglesias V (2018). Drug resistance in non-small cell lung cancer: a potential for NOTCH targeting?. Front. Oncol..

[25] Kang J (2013). Rhamnetin and cirsiliol induce radiosensitization and inhibition of epithelial-mesenchymal transition (EMT) by miR-34a-mediated suppression of Notch-1 expression in non-small cell lung cancer cell lines. J. Biol. Chem..

[26] Wang R (2018). iNOS promotes CD24+CD133+ liver cancer stem cell phenotype through a TACE/ADAM17-dependent Notch signaling pathway. Proc. Natl Acad. Sci. USA.

[27] Kato T, Hagiyama M, Ito A (2018). Renal ADAM10 and 17: their physiological and medical meanings. Front. Cell Dev. Biol..

[28] Li W (2019). ADAM17 promotes lymph node metastasis in gastric cancer via activation of the Notch and Wnt signaling pathways. Int. J. Mol. Med..

[29] Chen Z (2019). Hypomethylation-mediated activation of cancer/testis antigen KK-LC-1 facilitates hepatocellular carcinoma progression through activating the Notch1/Hes1 signalling. Cell Prolif..

[30] Fang S (2019). Lymphoid enhancer-binding factor-1 promotes stemness and poor differentiation of hepatocellular carcinoma by directly activating the NOTCH pathway. Oncogene.

[31] Feng F (2018). Pregnane X receptor mediates sorafenib resistance in advanced hepatocellular carcinoma. Biochim. Biophys. Acta Gen. Subj..

[32] Chen Y (2018). LINE-1 ORF-1p enhances the transcription factor activity of pregnenolone X receptor and promotes sorafenib resistance in hepatocellular carcinoma cells. Cancer Manag. Res..

[33] Wu M (2018). Triclosan treatment decreased the antitumor effect of sorafenib on hepatocellular carcinoma cells. Onco. Targets Ther..

[34] Gao X (2019). ARQ-197 enhances the antitumor effect of sorafenib in hepatocellular carcinoma cells via decelerating its intracellular clearance. Onco. Targets Ther..

[35] Chen Y (2015). MiRNA153 reduces effects of chemotherapeutic agents or small molecular kinase inhibitor in HCC cells. Curr. Cancer Drug Targets.

[36] Xie H (2018). A new apatinib microcrystal formulation enhances the effect of radiofrequency ablation treatment on hepatocellular carcinoma. Onco. Targets Ther..

[37] Wang. Y, Tang Z (2018). A novel long-sustaining system of apatinib for long-term inhibition of the proliferation of hepatocellular carcinoma cells. Onco. Targets Ther..

[38] Lu, Y. et al. LINE-1 ORF-1p functions as a novel androgen receptor co-activator and promotes the growth of human prostatic carcinoma cells. *Cell. Signal*. **25**, 479–489 (2013). 10.1016/j.cellsig.2012.11.004 23153584

[39] Yang Q (2013). LINE-1 ORF-1p functions as a novel HGF/ETS-1 signaling pathway co-activator and promotes the growth of MDA-MB-231 cell. Cell. Signal..

[40] Liang Y (2017). The EGFR/miR-338-3p/EYA2 axis controls breast tumor growth and lung metastasis. Cell Death Dis..

[41] Ji Q (2017). miR-216a inhibits osteosarcoma cell proliferation, invasion and metastasis by targeting CDK14. Cell Death Dis..

[42] Feng F (2013). Long interspersed nuclear element ORF-1 protein promotes proliferation and resistance to chemotherapy in hepatocellular carcinoma. World J. Gastroenterol..

[43] Li F (2018). Procaspase-3-activating compound 1 stabilizes hypoxia-inducible factor 1α and induces DNA damage by sequestering ferrous iron. Cell Death Dis..

[44] Guan F (2017). WX-132-18B, a novel microtubule inhibitor, exhibits promising anti-tumor effects. Oncotarget.

[45] Li J (2018). MicroRNA-140-3p enhances the sensitivity of hepatocellular carcinoma cells to sorafenib by targeting pregnenolone X receptor. Onco. Targets Ther..

[46] Fan Z (2019). PTK2 promotes cancer stem cell traits in hepatocellular carcinoma by activating Wnt/β-catenin signaling. Cancer Lett..

[47] Shao Z (2018). ETS-1 induces Sorafenib-resistance in hepatocellular carcinoma cells via regulating transcription factor activity of PXR. Pharmacol. Res..

[48] Li L (2018). Transcriptional regulation of the Warburg Effect in cancer by SIX1. Cancer Cell.

[49] Jia H (2016). Rhamnetin induces sensitization of hepatocellular carcinoma cells to a small molecular kinase inhibitor or chemotherapeutic agents. Biochim. Biophys. Acta.

[50] Zhang Y (2018). Novel ADAM-17 inhibitor ZLDI-8 enhances the in vitro and in vivo chemotherapeutic effects of Sorafenib on hepatocellular carcinoma cells. Cell Death Dis..

[51] Dang Q (2019). The γ-secretase inhibitor GSI-I interacts synergistically with the proteasome inhibitor bortezomib to induce ALK+ anaplastic large cell lymphoma cell apoptosis. Cell. Signal..

[52] Das A (2019). A novel triazole, NMK-T-057, induces autophagic cell death in breast cancer cells by inhibiting γ-secretase-mediated activation of Notch signaling. J. Biol. Chem..

[53] Xia W (2019). γ-Secretase and its modulators: twenty years and beyond. Neurosci. Lett..

[54] Wu S (2019). Bax inhibitor 1 is a γ-secretase-independent presenilin-binding protein. Proc. Natl Acad. Sci. USA.

[55] Balakumar P (2019). The renin-angiotensin-aldosterone system and epithelial-to-mesenchymal transition-induced renal abnormalities: mechanisms and therapeutic implications. Pharmacol. Res..

[56] Weng X (2019). PTPRB promotes metastasis of colorectal carcinoma via inducing epithelial-mesenchymal transition. Cell Death Dis..

[57] Li RH (2018). Long noncoding RNA ATB promotes the epithelial-mesenchymal transition by upregulating the miR-200c/Twist1 axe and predicts poor prognosis in breast cancer. Cell Death Dis..

[58] Mir N (2017). Epithelial-to-mesenchymal transition: a mediator of Sorafenib resistance in advanced hepatocellular carcinoma. Curr. Cancer Drug Targets.

[59] Chang L (2019). Targeting slug-mediated non-canonical activation of c-Met to overcome chemo-resistance in metastatic ovarian cancer cells. Acta Pharm. Sin. B.

[60] Fung SW (2019). The ATP-binding cassette transporter ABCF1 is a hepatic oncofetal protein that promotes chemoresistance, EMT and cancer stemness in hepatocellular carcinoma. Cancer Lett..

[61] Pinato DJ (2019). Integrated analysis of multiple receptor tyrosine kinases identifies Axl as a therapeutic target and mediator of resistance to sorafenib in hepatocellular carcinoma. Br. J. Cancer.

[62] Zhang PF (2019). LncRNA SNHG3 induces EMT and sorafenib resistance by modulating the miR-128/CD151 pathway in hepatocellular carcinoma. J. Cell Physiol..

[63] Niu L (2017). New insights into sorafenib resistance in hepatocellular carcinoma: responsible mechanisms and promising strategies. Biochim. Biophys. Acta Rev. Cancer.

[64] Li DD (2018). A novel inhibitor of ADAM17 sensitizes colorectal cancer cells to 5-Fluorouracil by reversing Notch and epithelial-mesenchymal transition in vitro and in vivo. Cell Prolif..

[65] An L (2017). Terfenadine combined with epirubicin impedes the chemo-resistant human non-small cell lung cancer both in vitro and in vivo through EMT and Notch reversal. Pharmacol. Res..

[66] Mollen EWJ (2018). Moving breast cancer therapy up a notch. Front. Oncol..

[67] Sosa Iglesias V (2018). Synergistic effects of NOTCH/γ-secretase inhibition and standard of care treatment modalities in non-small cell lung cancer cells. Front. Oncol..

[68] Zhang Z (2019). A patient-derived orthotopic xenograft (PDOX) nude-mouse model precisely identifies effective and ineffective therapies for recurrent leiomyosarcoma. Pharmacol. Res..

[69] Hou J (2017). A novel chemotherapeutic sensitivity-testing system based on collagen gel droplet embedded 3D-culture methods for hepatocellular carcinoma. BMC Cancer.

[70] Meng D (2019). A temperature-sensitive phase-change hydrogel of tamoxifen achieves the long-acting antitumor activation on breast cancer cells. Onco. Targets Ther..

[71] Meng D (2019). Effects of VEGFR1+ hematopoietic progenitor cells on pre-metastatic niche formation and in vivo metastasis of breast cancer cells. J. Cancer Res. Clin. Oncol..

[72] Meng D (2018). MicroRNA-645 targets urokinase plasminogen activator and decreases the invasive growth of MDA-MB-231 triple-negative breast cancer cells. Onco. Targets Ther..

[73] Li L (2017). miR-30a-5p suppresses breast tumor growth and metastasis through inhibition of LDHA-mediated Warburg effect. Cancer Lett..

[74] Kudo M (2018). Lenvatinib versus sorafenib in first-line treatment of patients with unresectable hepatocellular carcinoma: a randomised phase 3 non-inferiority trial. Lancet.

[75] Bruix J (2017). Regorafenib for patients with hepatocellular carcinoma who progressed on sorafenib treatment (RESORCE): a randomised, double-blind, placebo-controlled, phase 3 trial. Lancet.

[76] Xu J (2019). Anti-PD-1 antibody SHR-1210 combined with Apatinib for advanced hepatocellular carcinoma, gastric, or esophagogastric junction cancer: an open-label, dose escalation and expansion study. Clin. Cancer Res..

[77] Wang G (2019). Anlotinib, a novel small molecular tyrosine kinase inhibitor, suppresses growth and metastasis via dual blockade of VEGFR2 and MET in osteosarcoma. Int. J. Cancer.

