# Supplementary material for: MicroRNA-3163 targets ADAM-17 and enhances the sensitivity of hepatocellular carcinoma cells to molecular targeted agents
Source: Cell Death Dis. 2019 Oct 14;10(10):784. doi: 10.1038/s41419-019-2023-1 (PMC6791891; doi:10.1038/s41419-019-2023-1)
Supplement: Supplementary file 1 — Supplemental Table 2 [file 41419_2019_2023_MOESM1_ESM.doc]

**Supplemental Table 2 The primers used in the presence work**

| Targets | Primers | Sequences (5' to 3') |
| --- | --- | --- |
| Survivin | Forward Sequence | CCACTGAGAACGAGCCAGACTT |
|  | Reverse Sequence | GTATTACAGGCGTAAGCCACCG |
| cIAP-1 | Forward Sequence | CAGACACATGCAGCTCGAATGAG |
|  | Reverse Sequence | CACCTCAAGCCACCATCACAAC |
| cIAP-2 | Forward Sequence | GCTTTTGCTGTGATGGTGGACTC |
|  | Reverse Sequence | CTTGACGGATGAACTCCTGTCC |
| E-Cadherin | Forward Sequence | GCCTCCTGAAAAGAGAGTGGAAG |
|  | Reverse Sequence | TGGCAGTGTCTCTCCAAATCCG |
| N-Cadherin | Forward Sequence | CCTCCAGAGTTTACTGCCATGAC |
|  | Reverse Sequence | GTAGGATCTCCGCCACTGATTC |
| Vimentin | Forward Sequence | AGGCAAAGCAGGAGTCCACTGA |
|  | Reverse Sequence | ATCTGGCGTTCCAGGGACTCAT |
| ZEB1 | Forward Sequence | GGCATACACCTACTCAACTACGG |
|  | Reverse Sequence | TGGGCGGTGTAGAATCAGAGTC |
| Snail | Forward Sequence | TGCCCTCAAGATGCACATCCGA |
|  | Reverse Sequence | GGGACAGGAGAAGGGCTTCTC |
| Twist | Forward Sequence | GCCAGGTACATCGACTTCCTCT |
|  | Reverse Sequence | TCCATCCTCCAGACCGAGAAGG |
| GAPDH | Forward Sequence | GTCTCCTCTGACTTCAACAGCG |
|  | Reverse Sequence | ACCACCCTGTTGCTGTAGCCAA |
