# Supplementary figures and images for: MicroRNA-3163 targets ADAM-17 and enhances the sensitivity of hepatocellular carcinoma cells to molecular targeted agents
Source: Cell Death Dis. 2019 Oct 14;10(10):784. doi: 10.1038/s41419-019-2023-1 (PMC6791891; doi:10.1038/s41419-019-2023-1)

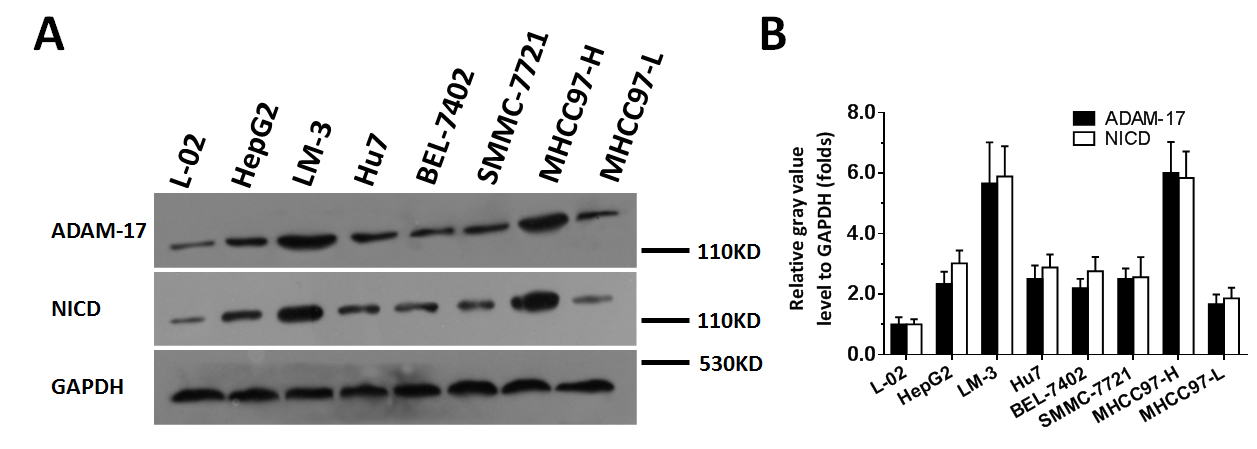

Supplement: Supplementary file 3 — Supplemental Figure 1 [file 41419_2019_2023_MOESM3_ESM.jpg]

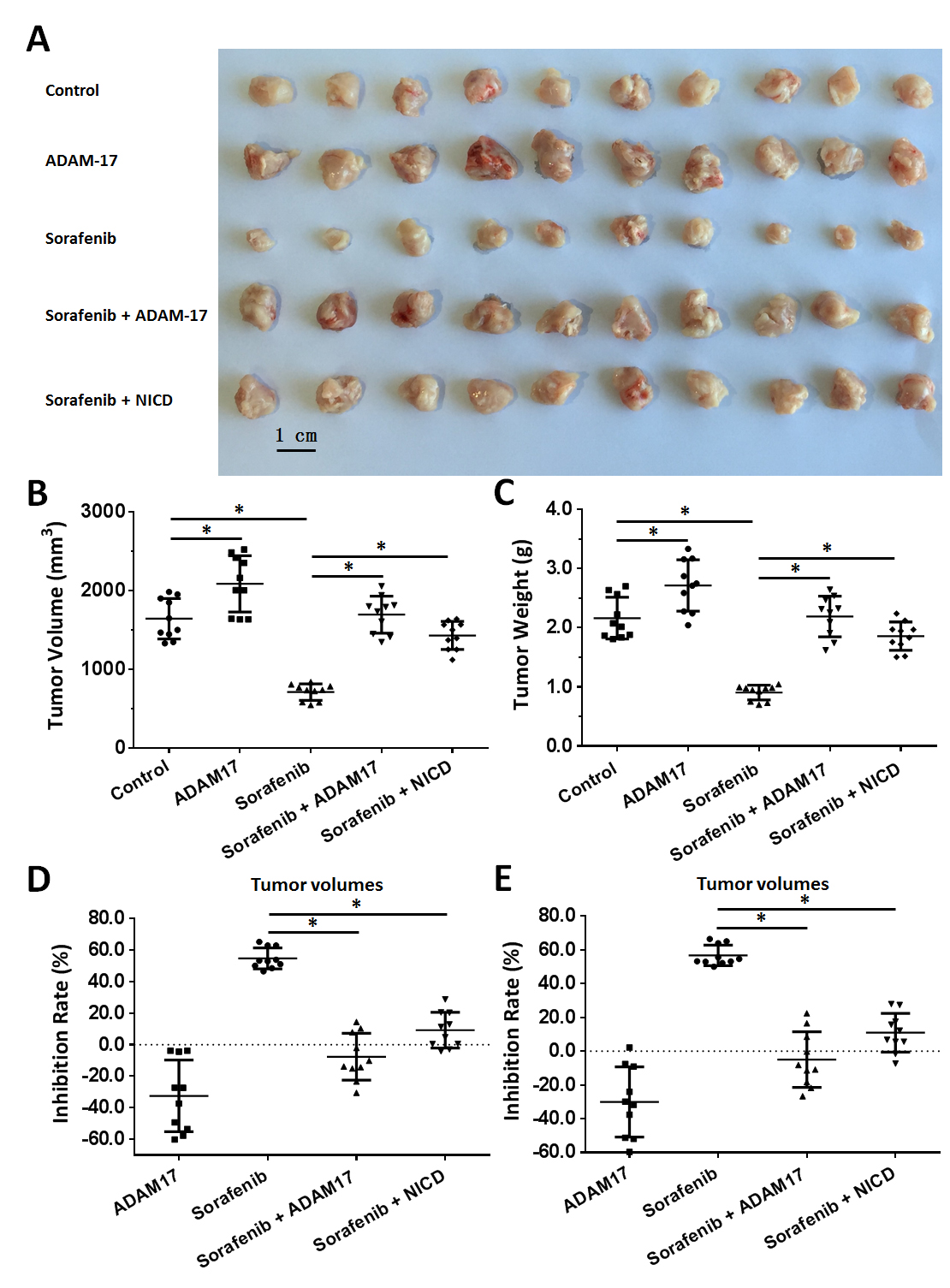

Supplement: Supplementary file 4 — Supplemental Figure 2 [file 41419_2019_2023_MOESM4_ESM.jpg]

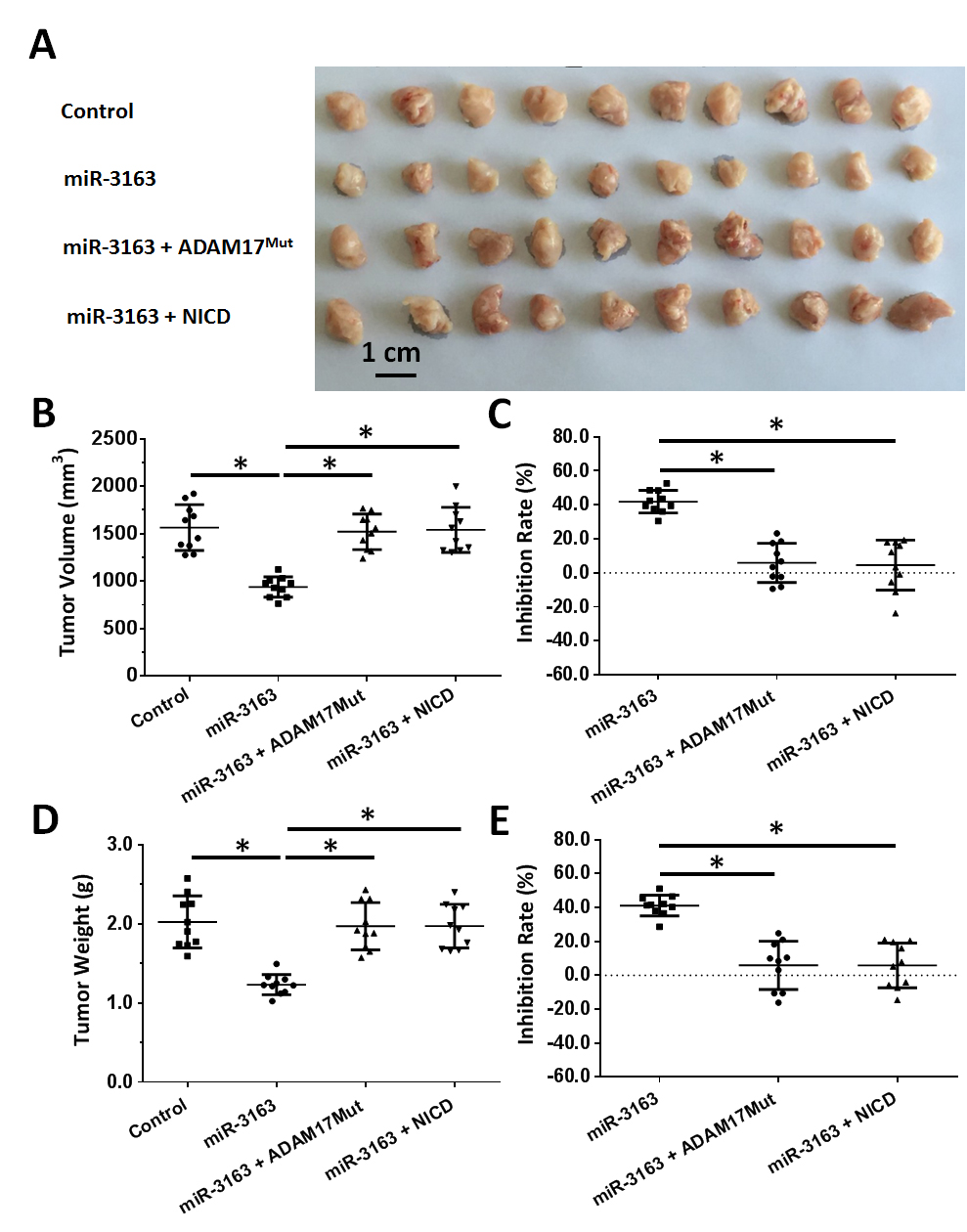

Supplement: Supplementary file 5 — Supplemental Figure 3 [file 41419_2019_2023_MOESM5_ESM.jpg]

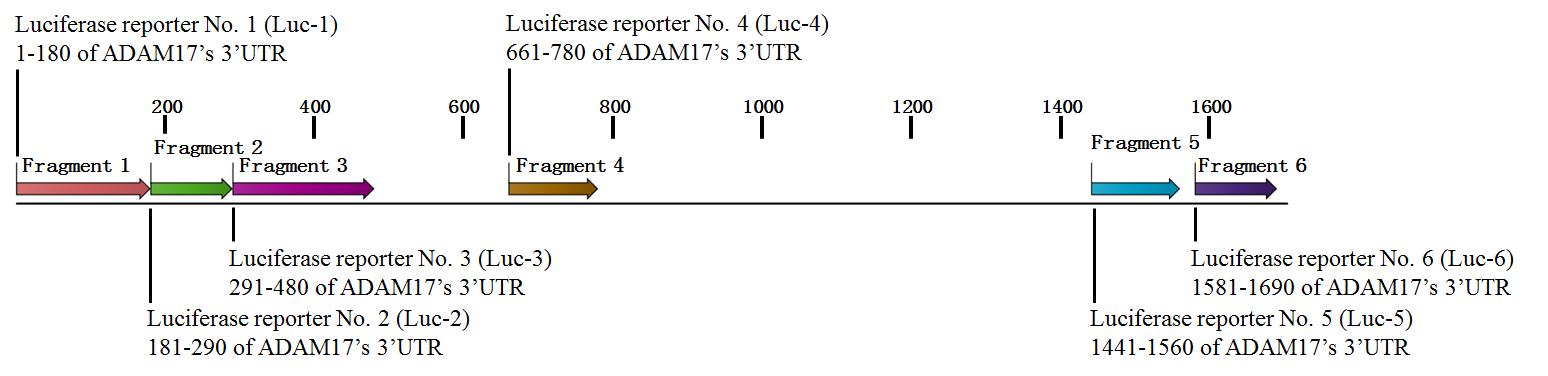

Supplement: Supplementary file 6 — Supplemental Fgure 4 [file 41419_2019_2023_MOESM6_ESM.jpg]

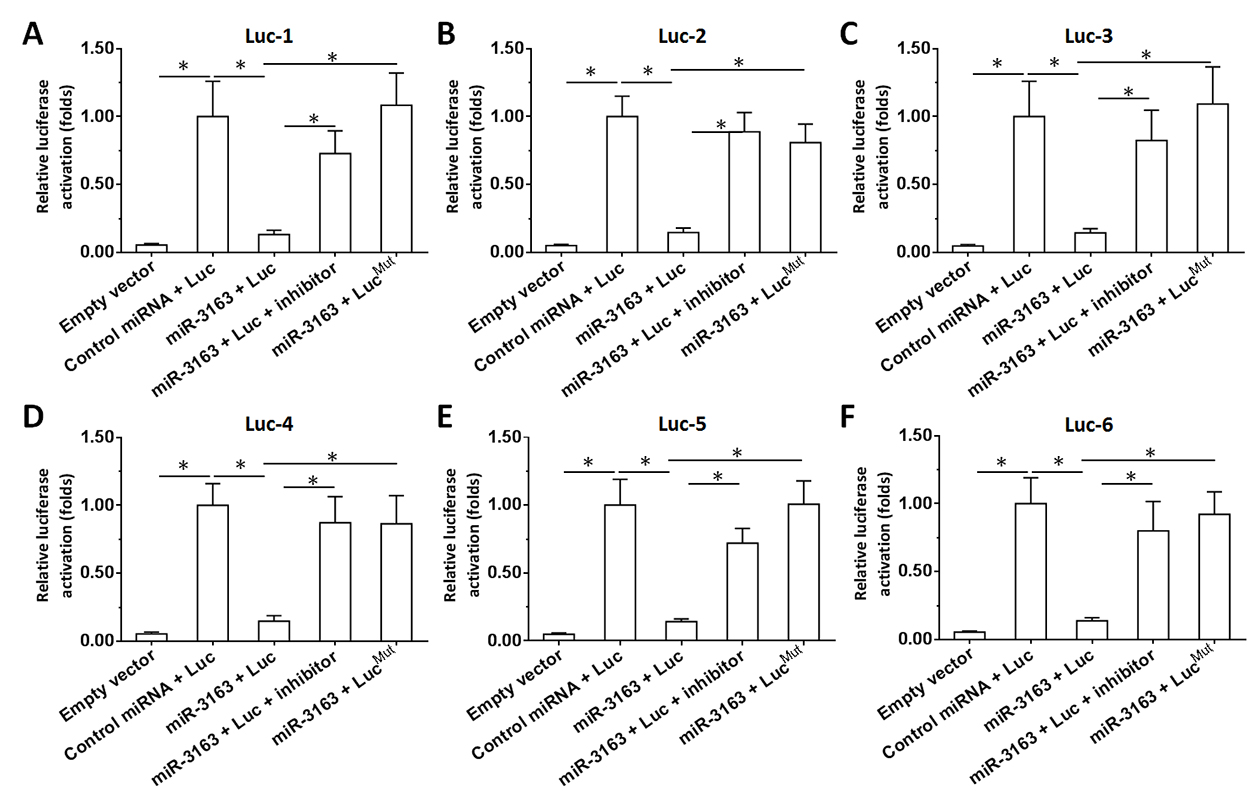

Supplement: Supplementary file 7 — Supplememental Figure 5 [file 41419_2019_2023_MOESM7_ESM.jpg]
