# Supplementary material for: MicroRNA-3163 targets ADAM-17 and enhances the sensitivity of hepatocellular carcinoma cells to molecular targeted agents
Source: Cell Death Dis. 2019 Oct 14;10(10):784. doi: 10.1038/s41419-019-2023-1 (PMC6791891; doi:10.1038/s41419-019-2023-1)
Supplement: Supplementary file 8 — Supplementary figures legends [file 41419_2019_2023_MOESM8_ESM.doc]

**Supplemental Figure 1** **The expression of ADAM-17 and NICD in hepatic cell lines**. Hepatic cell lines (L-02, HepG2, LM-3, Hu7, BEL-7402, SMMC-7721, MHCC97-H, or MHCC97-L) were cultured and harvested for western blotting analysis. The protein levels of ADAM-17 and NICD were examined using their antibodies. The results are shown as images of western blotting (A) or quantitative analysis (B).

**Supplemental Figure 2 ADAM-17 or NICD enhances the resistance of MHCC97-H cells to sorafenib**. MHCC97-H cells transfected with vectors (empty vector, ADAM-17, or NICD) were injected into nude mice to form subcutaneous tumors. The mice received oral administration of 2 mg/kg dose of sorafenib, and were harvested to collect tumor tissues. The results are shown as images of subcutaneous tumor tissues (A), tumor volumes (B), tumor weights (C), inhibition rates according to tumor volumes (D), or inhibition rates according to tumor weights (E). *P<0.05

**Supplemental Figure 3 miR-3163 inhibits the subcutaneous growth of MHCC97-H cells by targeting the ADAM-17/Notch signaling pathway**. MHCC97-H cells transfected with vectors (control miRNA, miR-3163, miR-3163 + ADAM-17^Mut^, or miR-3163 + NICD) were injected into nude mice to form subcutaneous tumors. The mice received oral administration of 2 mg/kg dose of sorafenib, and were harvested to collect tumor tissues. The results are shown as images of subcutaneous tumor tissues (A), tumor volumes (B), tumor weights (C), inhibition rates according to tumor volumes (D), or inhibition rates according to tumor weights (E). *P<0.05

**Supplemental Figure 4 Luciferase reporters of the 3’UTR sequences of ADAM-17 containing miR-3163 binding sites**. The 3’UTR sequences of ADAM-17 containing miR-3163 binding sites were cloned into pGL4.26 vectors. The results are shown as a schematic diagram mentioning the six luciferase reporters (1–180, 181–290, 291–480, 661–780, 1441–1560, or 1581–1690).

**Supplemental Figure 5 miR-3163 inhibits the activation of luciferase reporters of the 3’UTR sequences of ADAM-17 containing miR-3163 binding sites**. MHCC97-H cells transfected with vectors (control miRNA, luciferase reporters, miR-3163 + luciferase reporters, or miR-3163 + luciferase reporters with mutated miR-3163 binding sites) were analyzed in the luciferase experiments. The results are shown as mean±SD of the six luciferase reporters. *P<0.05
